# Genome-Wide Association Study for Nodule Traits in Guar

**DOI:** 10.3390/cimb47030151

**Published:** 2025-02-26

**Authors:** Shubham Malani, Waltram Ravelombola, Aurora Manley, Hanh Pham, Madeline Brown, Md. Mezanur Rahman

**Affiliations:** 1 Texas A&M AgriLife Research, 11708 Highway 70 South, Vernon, TX 76384, USA; 2 Soil and Crop Sciences, Texas A&M University, 370 Olsen Blvd., College Station, TX 77843, USA; 3 Texas A&M AgriLife Research, 1102 East Drew Street, Lubbock, TX 79403, USA; hanh.pham@ag.tamu.edu; 4 Department of Plant and Soil Science, Institute of Genomics for Crop Abiotic Stress Tolerance, Texas Tech University, Lubbock, TX 79409, USA

**Keywords:** guar, nodule, single nucleotide polymorphism, association, genetics

## Abstract

Guar [*Cyamopsis tetragonoloba* (L.) Taub] is a diploid legume crop cultivated for galactomannan (guar gum) extracted from the endosperm of the seed. Previous studies have suggested that nodulation of guar can be poor in field conditions; however, solid proof has yet to be provided. The objectives of this study were to conduct a genome-wide association study (GWAS) and to identify single nucleotide polymorphism (SNP) markers associated with nodules in guar. GWAS was performed on a total of 225 guar genotypes using 19,007 filtered SNPs. Tassel 5 was used to run five models: single marker regression (SMR), generalized mixed linear model with PCA as a covariate (GLM_PCA), generalized mixed linear model with Q matrix as a covariate (GLM_Q), mixed linear model with PCA and Kinship (K) as covariates (GLM_PCA + K), and mixed linear model with Q and K as covariates (MLM_Q + K). Across all statistical models, the results showed a total of 3, 2, 25, 7, 2, and 3 SNPs were associated with plant height, nodule number per plant, fresh nodule weight, dry nodule weight, fresh aboveground plant biomass, and dry aboveground plant biomass. These SNPs could be used as a tool to select for better nodule traits in guar.

## 1. Introduction

Guar [*Cyamopsis tetragonoloba* (L.) Taub] is a diploid (2n = 2x = 14) legume crop [1] and is also known as cluster bean, gauri, chavlikayi, or kutti [2]. It is grown in several semiarid regions of the world including India, Pakistan, the U.S., and South Africa [3]. Guar production shows great potential in other parts of the world with a hot environment and limited water availability [4]. Guar has historically been used as a crop for green manure, food, and forage [5]. The large concentration of galactomannans, known as guar gum, which accumulates in the endosperm of mature guar seeds is still the main reason for guar cultivation. Additionally, guar gum functions as a hydrocolloid, which is a plant polymer composed of individual monosaccharides (galactose) producing gelatinous mixtures that are resistant to freezing when dissolved in water or other liquids and have been used in many food products, pharmaceuticals, cosmetics, explosives, meat products, pet foods, and textile industry [5]. A large amount of guar gum is used by the gas and oil industry for more efficient hydraulic fracturing [6,7,8,9]. Guar gum is a gum product that has the benefits of being affordable, non-toxic, renewable, and biodegradable [10].

Legumes can form a symbiotic association with rhizobia bacteria that fix atmospheric nitrogen, allowing them to thrive in nitrogen-deficient environments [11]. Few studies on biological nitrogen fixation (BNF) of guar have been reported in the literature, and researchers have noted that guar nodulation may be poor in field settings [5,12]. Another study examined the nodule features and nitrogenase activity in guar under drought stress [13]. They found that the number of nodules was unaffected by drought. On the other hand, nodule fresh weight and nitrogenase activity were shown to decrease in response to drought stress. Inoculated guar produced 36% more nodules on average per plant than non-inoculated guar of the same type [1]. Nodule mass is slightly controlled by both genetic and environmental factors, and nodule number per plant is mostly impacted by non-genetic variables such as soil moisture, soil nitrogen (N), soil type, and Rhizobium inoculation [10].

As plant breeding developed, many strategies were used to address agricultural issues, such as the development of molecular marker systems [14]. Molecular genetic markers link the genotype behind observable phenotypes and explain genetic variations [15]. The development of molecular markers to select higher levels of N-fixing capacity may be possible by discovering important genetic variables linked to nodulation and the physiological response in a diverse collection of genotypes [16]. Single nucleotide polymorphisms (SNPs) are molecular markers used in plant breeding and genetics [17]. The use of SNPs in genetic mapping permits the establishment of higher mapping resolutions and provides denser genetic maps [18]. The use of SNP markers to screen for desired nodule traits can be a solution to the rapid development of germplasm with improved nodule traits. Molecular markers such as SNPs have not been used in previous studies to better understand the genetic basis of nodulation and symbiotic nitrogen fixation in the major legume crop guar. Therefore, the objectives of this study were to conduct a genome-wide association study (GWAS) and to identify single nucleotide polymorphism (SNP) markers associated with nodules in guar. We hypothesized that GWAS can be used to accurately identify SNPs and candidate genes related to nodule traits in guar.

## 2. Materials and Methods

### 2.1. Plant Materials, Phenotyping, and Genotyping

A total of 225 guar accessions from the United States Department of Agriculture (USDA) and three commercial varieties including ‘Kinman’, ‘Lewis’, and ‘Santa Cruz’ were used for the genome-wide association study. The guar accessions were evaluated for nodule traits including plant height, nodule number per plant, nodule diameter, fresh nodule weight, dry nodule weight, and fresh and dry aboveground plant biomass. The phenotypic data were described in our previous study [19]. The guar panel was genotyped using a total of 19,007 filtered single nucleotide polymorphisms (SNPs) that were obtained from genotyping-by-sequencing (GBS). A population structure analysis of the guar panel from our previous study showed optimal delta Ks at K = 3 and K = 2 for this panel [20]. Therefore, Q matrices corresponding to K = 3 and K = 2 were used as covariates for the genome-wide association study (GWAS).

### 2.2. Genome-Wide Association Study (GWAS)

GWAS was conducted in Tassel 5 using the following models: single marker regression (SMR), generalized mixed linear model using PCA as a covariate (GLM_PCA), generalized mixed linear model using Q matrix as a covariate (GLM_Q), mixed linear model using both PCA and Kinship (K) as covariates (GLM_PCA + K), and mixed linear model using both Q and K as covariates (GLM_Q + K). Both PCA and K matrices were estimated using Tassel 5. SNPs with a LOD (logarithm of the odds) value > 3.5 were considered significant SNPs associated with the traits of interest [18]. LOD scores were used to assess the possibility that a certain genomic region was linked to a desired trait. Several statistical methods were used in the genome-wide association study (GWAS) to find QTLs linked to nodule traits including plant height, nodule number, nodule diameter, fresh and dry nodule weight, and fresh and dry plant biomass in guar. To reduce false positive connections, more conservative models were used, such as the mixed linear model (MLM) and general linear model (GLM). On the other hand, less conservative models with a higher chance of false positives, such as SMR and generalized linear model (PCA), have a stronger ability to identify genuine QTLs. A compromise between sensitivity to detect lesser QTL effects and accuracy to identify major markers may be achieved by utilizing several models with different stringencies. Consistently identified SNPs through different models provide better chances of them being less statistical artifacts. Overall, the use of different statistical models gives more robust results. For each trait, a candidate gene search was conducted using a 20 kb region spanning an SNP that was consistent across different models. Annotated genes with biological functions pertaining to nodulations and plant growth were selected.

## 3. Results

### 3.1. Plant Height

**Figure 1:**
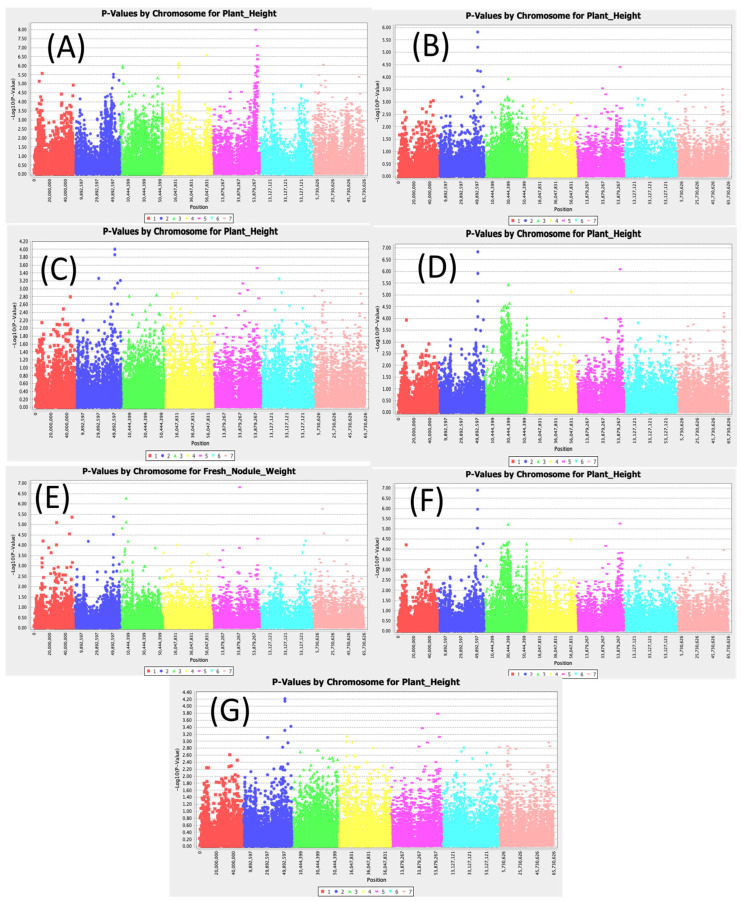
Manhattan plots for plant height: (**A**) single marker regression (SMR), (**B**) generalized linear model (PCA), (**C**) mixed linear model (PCA), (**D**) generalized linear model (Q3), (**E**) mixed linear model (Q3), (**F**) generalized linear model (Q2), and (**G**) mixed linear model (Q2).

A total of 256 significant SNPs were found to be associated with plant height (Figure 1). Distinct associated SNPs were revealed by different models, including the least conservative SMR model and the most conservative MLM Q3+K models. The SNPs Chr2_51078296, Chr2_51010513, and Chr5_55996909 were consistently found to be significant in all models. Population structure and kinship were controlled for in the MLM Q3+K model, which found LOD scores of 3.85, 4.02, and 4.09 for the SNPs Chr2_51078296, Chr2_51010513, and Chr5_55996909, respectively. These effects were further supported by the SMR model, with significantly higher LOD scores of 5.53, 5.37, and 7.99 for the SNPs Chr2_51078296, Chr2_51010513, and Chr5_55996909, respectively. The most significant amount of phenotypic variation was explained by the SNP Chr5_55996909 having an R2 value up to 15.40 (Table S1), suggesting that this SNP had the highest correlation with plant height in this germplasm pool. When combined, these data offer strong support from complementary models for the substantial relationships between the SNPs Chr2_51078296, Chr2_51010513, and Chr5_55996909 and plant height variation in guar, with Chr5_55996909 having the largest effect.

### 3.2. Nodule Number per Plant

**Figure 2:**
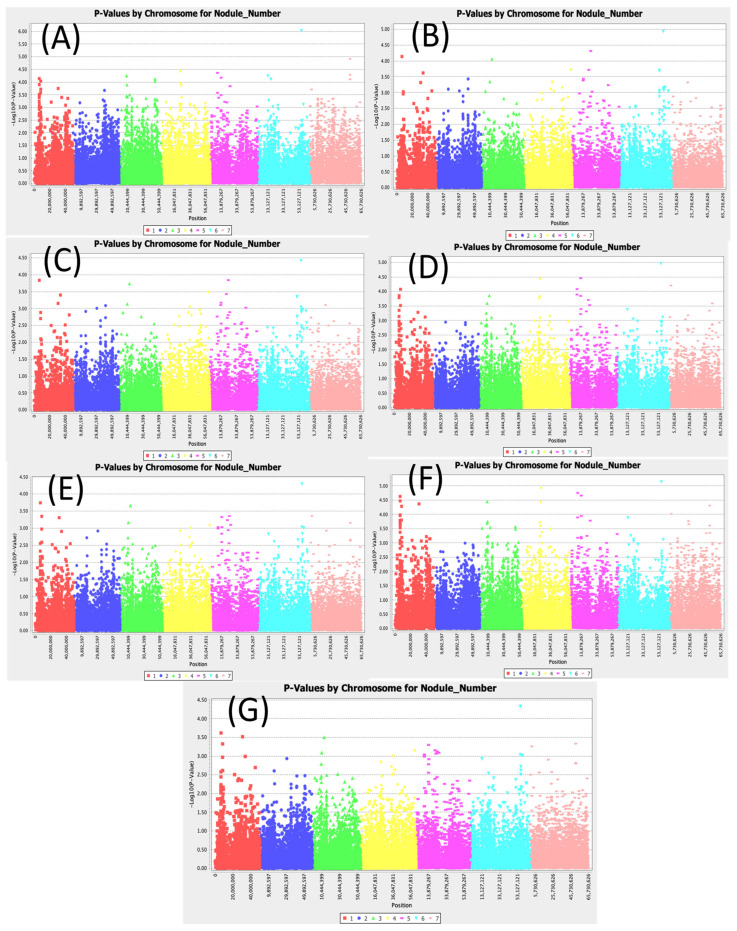
Manhattan plots for nodule number: (**A**) single marker regression (SMR), (**B**) generalized linear model (PCA), (**C**) mixed linear model (PCA), (**D**) generalized linear model (Q3), (**E**) mixed linear model (Q3), (**F**) generalized linear model (Q2), and (**G**) mixed linear model (Q2).

A total of 34 SNPs were found to be associated with nodule number per plant (Figure 2). Two SNPs consisting of Chr3_13052718 and Chr6_56449898 showed significant association with nodule number across all models. LOD values for the MLM (Q3+K) model were 3.66 and 4.30 for the SNPs Chr3_13052718 and Chr6_56449898, respectively. For the SMR model, which is the least conservative model, LOD values were 3.5 and 6.02 for the SNPs Chr3_13052718 and Chr6_56449898, respectively (Table S2). The SNP Chr6_56449898 had the highest LOD value across all models with an R2 value up to 11.86%.

### 3.3. Nodule Diameter

**Figure 3:**
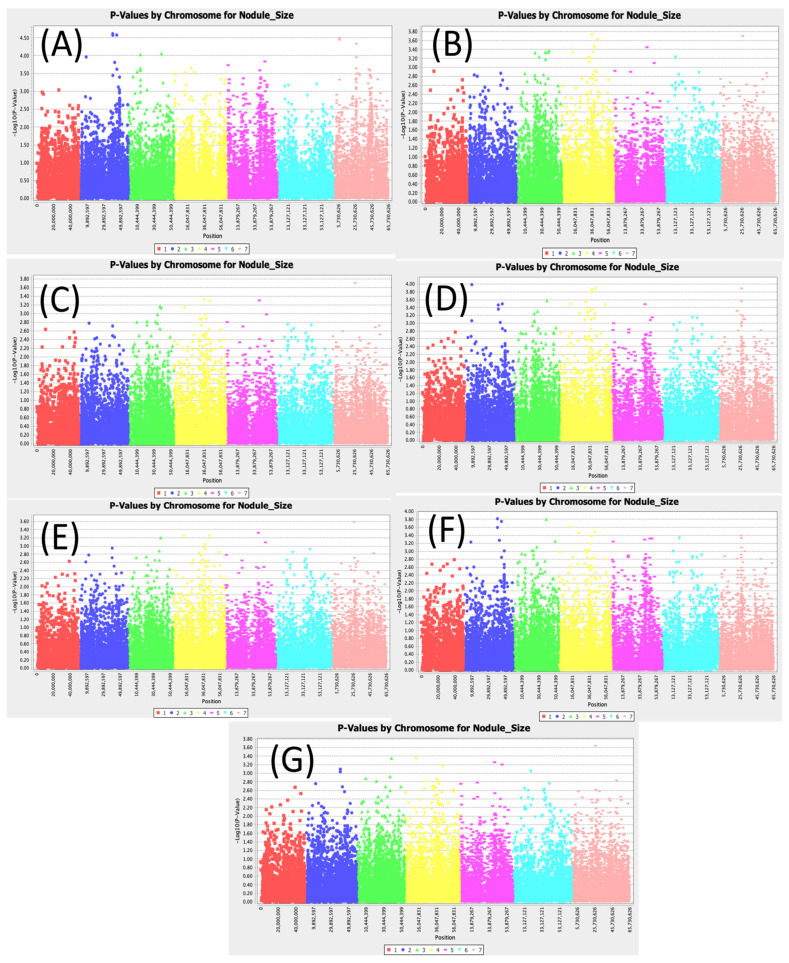
Manhattan plots for nodule diameter: (**A**) single marker regression (SMR), (**B**) generalized linear model (PCA), (**C**) mixed linear model (PCA), (**D**) generalized linear model (Q3), (**E**) mixed linear model (Q3), (**F**) generalized linear model (Q2), and (**G**) mixed linear model (Q2).

Table S3 shows 30 significant SNPs associated with nodule diameter across all models (Figure 3). Interestingly, no SNPs were significant in all models, indicating the genetic complexity of this trait and the possibility that this trait can be influenced by multiple genes with small effects. However, across all MLM models, the SNP Chr7_26646738 showed the highest LOD value ranging from 3.58 to 3.71. The SNP Chr3_39258868 had the highest LOD values of 4.05, 3.58, and 3.80 for the SMR, GLM (Q3), and GLM (Q2), respectively.

### 3.4. Fresh Nodule Weight

**Figure 4:**
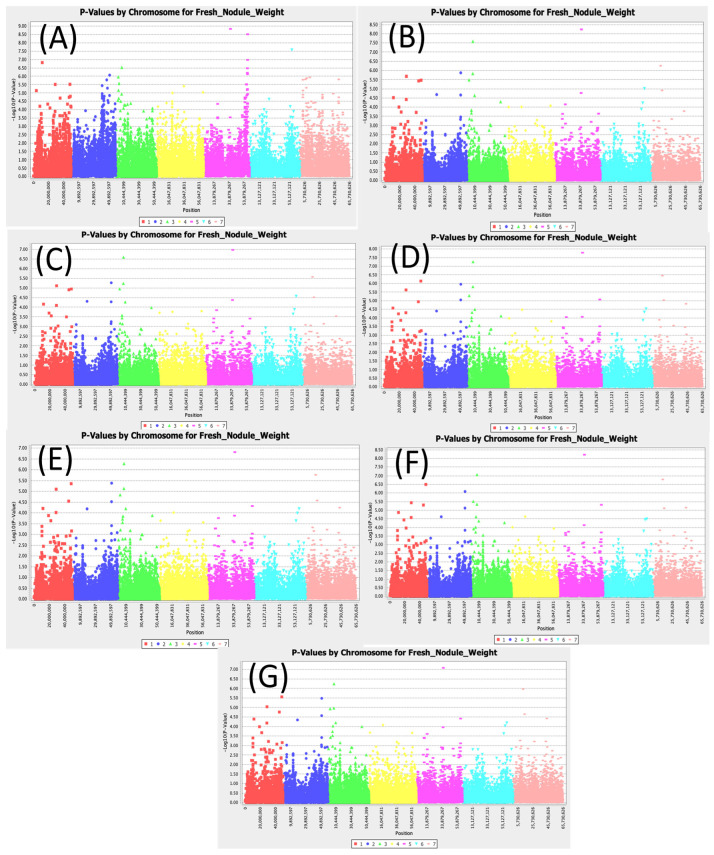
Manhattan plots for nodule fresh weight: (**A**) single marker regression (SMR), (**B**) generalized linear model (PCA), (**C**) mixed linear model (PCA), (**D**) generalized linear model (Q3), (**E**) mixed linear model (Q3), (**F**) generalized linear model (Q2), and (**G**) mixed linear model (Q2).

Table S4 shows 212 SNPs that were found to be significantly associated with fresh nodule weight. A total of 25 SNPs were significant across all models used for this study (Figure 4). The LOD values for the most conservative model MLM (Q3+K) were found for 25 different SNPs. The highest LOD values for the top five SNPs were 6.81, 6.28, 5.77, 5.38, and 5.36 for the SNPs Chr5_35619658, Chr3_7773837, Chr7_12878334, Chr2_51078296, and Chr1_48466801, respectively (Table S4). The LOD values for less conservative model SMR were also found for 25 different SNPs. The highest LOD values for the top five SNPs were 8.84, 7.58, 6.82, 6.53, and 6.07 for the SNPs Chr5_35619658, Chr6_56449898, Chr1_11676109, Chr3_7773837, and Chr2_51078296, respectively. The SNP Chr5_35619658 had the highest LOD values in all models and had an R2 value of up to 16.82.

### 3.5. Dry Nodule Weight

**Figure 5:**
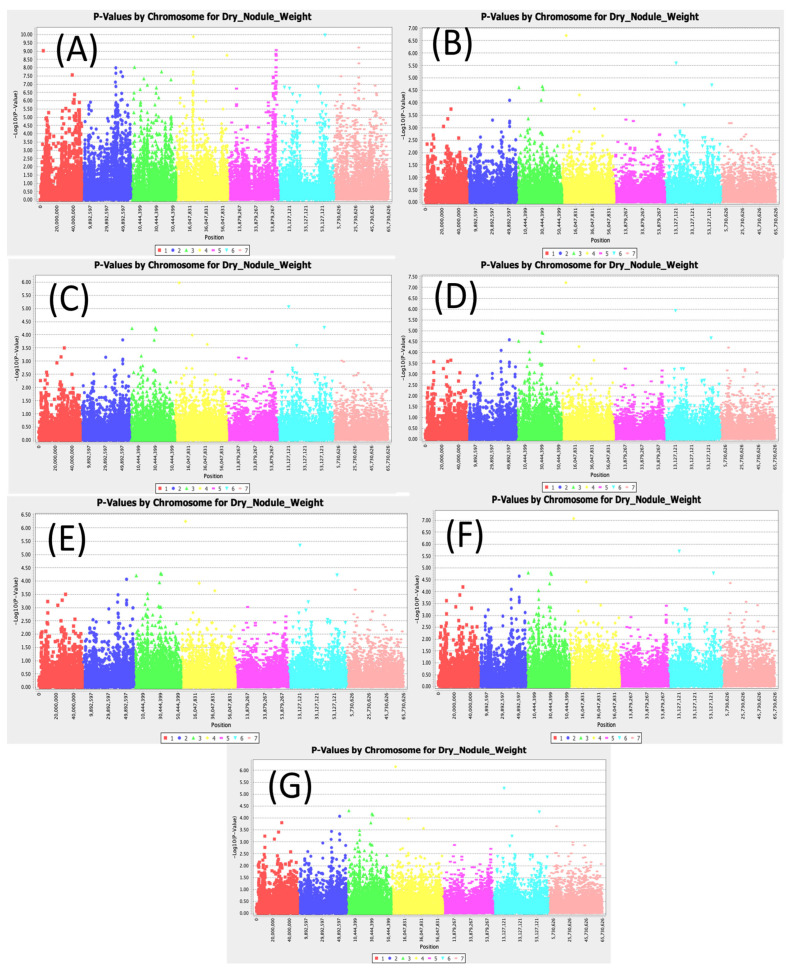
Manhattan plots for nodule dry weight: (**A**) single marker regression (SMR), (**B**) generalized linear model (PCA), (**C**) mixed linear model (PCA), (**D**) generalized linear model (Q3), (**E**) mixed linear model (Q3), (**F**) generalized linear model (Q2), and (**G**) mixed linear model (Q2).

GWAS identified 732 SNPs significantly associated with dry nodule weight in this study (Table S5). The SNPs Chr2_50961410, Chr3_29368549, Chr3_30748062, Chr3_31743839, Chr4_20715064, Chr4_4985600, Chr6_13748571, Chr6_56449898 SNPs were found to be significantly associated with dry nodule weight across models (Figure 5). The most conservative model MLM (Q3+K) showed that the SNPs Chr2_50961410, Chr3_29368549, Chr3_30748062, Chr3_31743839, Chr4_20715064, Chr4_4985600, Chr6_13748571, and Chr6_56449898 had the highest LOD scores of 4.07, 3.95, 4.29, 4.26, 3.92, 6.24, 5.35, and 4.23, respectively. For MLM (Q3+K), the SNP Chr4_4985600 had the highest LOD value and an R2 of 12.98%. For the SMR model, the SNPs Chr2_50961410, Chr3_29368549, Chr3_30748062, Chr3_31743839, Chr4_20715064, Chr4_4985600, Chr6_13748571, and Chr6_56449898 had the highest LOD values of 4.00 3.55, 4.04, 3.95, 9.87, 6.17, 6.74, and 9.97, respectively. For SMR, the SNP Chr6_56449898 had the highest LOD and R2 value of 18.83.

### 3.6. Fresh Aboveground Biomass

**Figure 6:**
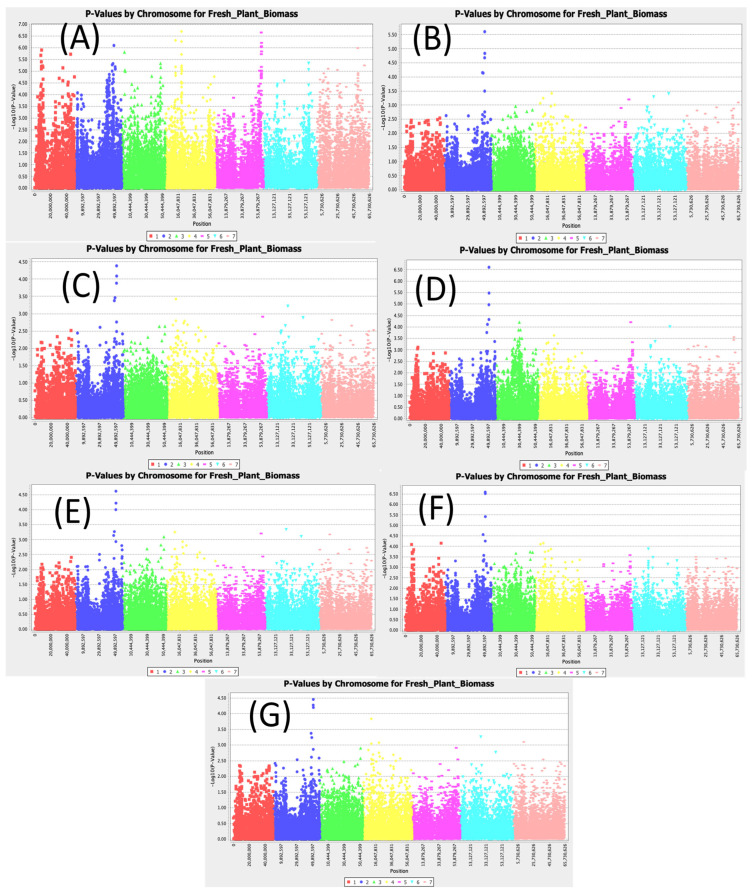
Manhattan plots for aboveground fresh biomass: (**A**) single marker regression (SMR), (**B**) generalized linear model (PCA), (**C**) mixed linear model (PCA), (**D**) generalized linear model (Q3), (**E**) mixed linear model (Q3), (**F**) generalized linear model (Q2), and (**G**) mixed linear model (Q2).

Table S6 displays the significant SNPs associated with fresh aboveground plant biomass. Three significant SNPs located on chromosome 2 with Chr2_50910112, Chr2_51010513, and Chr2_51078296 were consistent across different models (Figure 6). For these three SNPs, the LOD values under the most conservative MLM (Q3+K) model were 4.00, 4.62, and 4.22 for the SNPs Chr2_50910112, Chr2_51010513, and Chr2_51078296, respectively. For the SMR model, the LOD values were 5.19, 5.10, and 5.09 for the SNPs Chr2_50910112, Chr2_51010513, and Chr2_51078296, respectively. The SNP Chr2_51010513 had the highest LOD value in the MLM (Q3+K) model and the second highest in the SMR model with an R2 value of up to 10.09%.

### 3.7. Dry Aboveground Biomass

**Figure 7:**
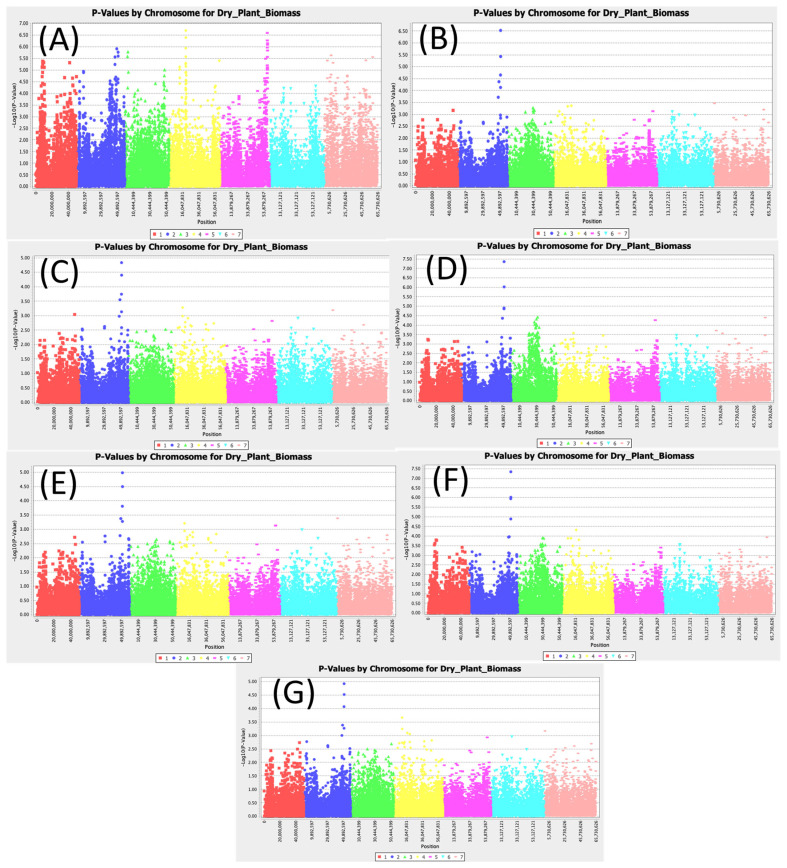
Manhattan plots for aboveground dry biomass: (**A**) single marker regression (SMR), (**B**) generalized linear model (PCA), (**C**) mixed linear model (PCA), (**D**) generalized linear model (Q3), (**E**) mixed linear model (Q3), (**F**) generalized linear model (Q2), and (**G**) mixed linear model (Q2).

Table S7 shows the significant SNPs associated with dry aboveground plant biomass. Dry aboveground plant biomass had the same three significant SNPs (Chr2_50910112, Chr2_51010513, and Chr2_51078296) as fresh aboveground plant biomass across all models (Figure 7). The LOD values for the SNPs Chr2_50910112, Chr2_51010513, and Chr2_51078296 were 3.81, 4.98, and 4.50, respectively, using the MLM (Q3+K) model. Conversely, the LOD values for the SNPs Chr2_50910112, Chr2_51010513, and Chr2_51078296 were 4.61, 5.77, and 5.60, respectively, according to the SMR model, which were the least conservative of all the models assessed. The SNP Chr2_51010513 had a greater LOD value across all models, with an R2 value as high as 11.32%.

### 3.8. Overlapping SNPs Between Traits

Significant interrelationships among the evaluated phenotypes and SNPs were found, indicating the possibility of pleiotropic genetic effects on trait variation. The SNP Chr2_51078296 was significantly associated with plant height, fresh and dry aboveground plant biomass, and fresh nodule weight. Likewise, the SNP Chr2_51010513 was associated with plant height and fresh/dry aboveground plant biomass. The SNP Chr6_56449898 was associated with nodule number and fresh/dry nodule weight.

### 3.9. Candidate Genes

Table 1 shows the candidate genes found in the vicinity of the most significant SNPs that were consistent across all models. Two candidate genes consisting of *Cyate.05.100546* and *Cyate.05.100724* were found for plant height. *Cyate.05.100546* and *Cyate.05.100724* encode for transcription factor NF-Y alpha-related and serine/threonine protein kinase, respectively. *Cyate.06.305325* was associated with nodule number per plant and encodes for vacuolar iron-like protein. This candidate gene was also found in the vicinity of the SNP associated with fresh and nodule dry weight. In addition to *Cyate.06.305325*, another candidate gene *Cyate.05.100456*, encoding for a nodulin protein, was found for fresh nodule weight. Candidate gene *Cyate.06.443643* was identified for nodule diameter. *Cyate.02.301545* was found as a potential candidate gene for both fresh and dry aboveground biomass. Another candidate gene *Cyate.02.453004* encoding for calcium-binding protein was found for plant height, fresh and dry aboveground plant biomass, and fresh nodule weight.

## 4. Discussion

We conducted a genome-wide association analysis (GWAS) to determine the genomic regions linked to guar nodule traits. Guar symbiotically associates with rhizobia bacteria, resulting in the formation of root nodules where nitrogen from the atmosphere is transformed into ammonia for plant growth [4]. Enhancing the nodule’s physical and morphological characteristics, including quantity, diameter, and weight, can improve guar productivity and nitrogen uptake. However, the genetic mechanism for guar nodule features remains poorly understood. Through GWAS, a total of 1849 SNP markers were identified to be associated with seven traits including plant height, nodule number per plant, nodule diameter, nodule fresh and dry weight, and aboveground fresh and dry biomass. These markers offer valuable prospects for future characterization of genes regulating nodulation in guar, as well as for marker-assisted selection. These results showed that genome-wide association studies can contribute to understanding the genetic architecture of complex traits.

On chromosomes 2, 5, and 6, several genomic regions with clusters of significant SNPs were found. A cluster of eight SNPs was linked to all seven attributes on chromosome 2, which was shown to be the most relevant location. This suggests that significant loci controlling guar nodule growth are located on chromosome 2. There have been reports of shared QTLs between symbiotic characteristics in other legumes, such as Medicago (*Medicago truncatula* Gaertn.) and cowpea (*Vigna unguiculata* (L.) Walp) [18,21]. We also discovered many SNPs that are particularly linked to individual traits, suggesting separate genetic control for these traits. Similar findings with both common and different QTLs for nodule number and mass were observed in narrow-leafed lupin (*Lupinus angustifolius* L.) [22].

Overall, decreasing values in LOD and R_2_ were found from the least most conservative models (SMR to MLM). These outcomes were anticipated because each of the four models included a different set of parameters intended to reduce false positives [23]. The original model was called single marker regression (SMR), which only included genotypes and phenotypes (SNPs). The second model was a general linear model (GLM) that used the Q matrix from the population structure analysis, the genotypic data (SNPs), and the phenotypic data [18]. A mixed linear model (MLM) comprising the phenotype, genotype, Q matrix, and Kinship K from TASSEL 5 was the third one where K had a random effect [24]. Robust SNP markers were identified in this research due to the consistency of several significantly reported SNPs across models despite the differences in the parameters utilized in each model. Our findings highlight the ability of GWAS to analyze the genetics underpinning guar nodule features that are complicated. We found markers without misleading connections by taking relatedness and population structure into consideration. Prospective fine-mapping endeavors may enhance the resolution of QTL areas and candidate gene discovery. Similar wide QTL areas needing validation have been found by nodule GWAS in soybean (*Glycine max* (L.) Merr.) and *Medicago* crops [21,24].

In this study, we found that the SNP Chr2_51078296 was associated with plant height, fresh and dry aboveground plant biomass, and fresh nodule weight in guar. The production of biomass per plant is directly correlated with plant height because, as plant height grows, so does plant biomass [25]. The aboveground plant biomass of guar is highly correlated with nodule weight [10]. The genetic area containing the Chr2_51078296 marker contains a quantitative trait locus that influences guar nodulation capacity, as evidenced by the substantial relationship that was found. Multi-trait SNP locus provides a promising target for further functional validation to elucidate the underlying mechanism coupling variation in height and biomass accumulation. Only a few studies on genome-wide association that examine different phenotypic traits have been reported in guar [18,26]. There is a very limited genetic study on guar nodulation available and this research may be the first study to accomplish this goal. These SNPs can be a useful tool for guar breeders to choose plants with better nodule traits. These molecular markers might help breeding programs quickly identify plants that are better for nodulation. Furthermore, the findings may significantly contribute to our understanding of the genetic architecture controlling nodulation in guar. Multiple candidate genes with functional annotations relevant to nodule traits were identified in this study. Two candidate genes encoding for transcription factor NF-Y alpha-related and serine/threonine protein kinase were identified for plant height. These proteins have been reported to be involved in plant growth and development in other crops such as soybean and *Arabidopsis* [21]. Candidate gene encoding for vacuolar iron transporter was found to be associated with nodule number per plant, and fresh and dry nodule weight. Vacuolar iron transporter was demonstrated to be involved in symbiotic nitrogen fixation in soybean nodules [16]. These findings suggest that the SNPs identified in this study are strongly associated with nodule-related traits in guar. A candidate gene encoding for a nodulin protein was also found for fresh nodule diameter. Nodulin protein is involved in atmospheric nitrogen fixation in plants and plays metabolic and structural roles within plant nodules [16]. In addition, a gene encoding for purple acid phosphatase regulating plant growth [21], was also found to be associated with fresh and dry aboveground weight in guar. These results show that the candidate genes reported in this study have functional annotations that are directly related to plant growth and nodule traits. To fully understand the genetic pathways driving guar nodulation, additional research is necessary.

## 5. Conclusions

Different SNPs associated with many nodule traits were identified through a genome-wide association study in guar. There were multiple SNPs found associated with different traits in guar nodulation. The SNPs Chr2_51078296 and Chr2_51010513 were highly associated with nodule traits and other phenotypes such as plant height and biomass. Additionally, SNP Chr6_56449898 was highly associated with nodule number and weight. To the best of our knowledge, this is one of the first studies to reveal SNP markers for guar nodule traits. These SNPs may be a useful tool in marker-assisted selection to improve guar genotype nodulation. To more fully utilize these SNPs and accelerate genetic gain for guar nodule traits, further validation, fine-mapping, and integration into multivariate genomic selection models are required. Additional transcriptomic and gene-editing approaches can also further validate the functions of the candidate genes reported in this study. Molecular markers can be designed using SNP information with candidate genes directly related to nodule functions and structure. These molecular markers can be used in a marker-assisted breeding approach to improve nodule traits in guar and other legumes.

## Figures and Tables

**Table 1:** Candidate genes and functional annotations.

Traits	SNP	Chromosome	Physical Position (bp)	Annotated Genes	Functional Annotations
Plant_Height	Chr5_55996909	5	55996909	*Cyate.05.100546*	Transcription factor NF-Y alpha-related
				*Cyate.05.100724*	Serine/threonine protein kinase
Nodule number per plant	Chr6_56449898	6	56449898	*Cyate.06.305325*	Vacuolar iron transporter-like
Nodule diameter	Chr7_26646738	7	26646738	*Cyate.06.443643*	Response to symbiont
Fresh nodule weight	Chr5_35619658	5	35619658	*Cyate.05.100456*	Nodulin protein
Dry nodule weight	Chr6_56449898	6	56449898	*Cyate.06.305325*	Vacuolar iron transporter-like
Fresh biomass	Chr2_51010513	2	51010513	*Cyate.02.301545*	Purple acid phosphatase
Dry biomass	Chr2_51010513	2	51010513	*Cyate.02.301545*	Purple acid phosphatase
Plant height, fresh and dry aboveground plant biomass, and fresh nodule weight	Chr2_51078296	2	51078296	*Cyate.02.453004*	Calcium-binding protein
Plant height, fresh and dry aboveground plant biomass	Chr2_51010513	2	51010513	*Cyate.02.301545*	Purple acid phosphatase
Nodule number, and fresh and dry nodule weight	Chr6_56449898	6	56449898	*Cyate.06.305325*	Vacuolar iron transporter-like

## Data Availability

Data are within the article.

## Supplementary Materials

The following supporting information can be downloaded at: https://www.mdpi.com/article/10.3390/cimb47030151/s1.

## References

[1] Ravelombola W., Manley A., Adams C., Trostle C., Ale S., Shi A., Cason J. (2021). Genetic and Genomic Resources in Guar: A Review. Euphytica.

[2] Kumar D., Rodge A. (2012). Status, Scope and Strategies of Arid Legumes Research in India-A Review. J. Food Legumes.

[3] El-Sawah A.M., El-Keblawy A., Ali D.F.I., Ibrahim H.M., El-Sheikh M.A., Sharma A., Hamoud Y.A., Shaghaleh H., Brestic M., Skalicky A. (2021). Arbuscular Mycorrhizal Fungi and Plant Growth-Promoting Rhizobacteria Enhance Soil Key Enzymes, Plant Growth, Seed Yield, and Qualitative Attributes of Guar. Agriculture.

[4] Gresta F., Trostle C., Sortino O., Santonoceto C., Avola G. (2019). Rhizobium Inoculation and Phosphate Fertilization Effects on Productive and Qualitative Traits of Guar (*Cyamopsis tetragonoloba* (L.) Taub.). Ind. Crop. Prod..

[5] Abidi N., Liyanage S., Auld D., Imel R., Norman L., Grover K., Angadi S., Singla S., Trostle C. (2021). Challenges and Opportunities for Increasing Guar Production in the United States to Support Unconventional Oil and Gas Production. Hydraulic Fracturing Impacts and Technologies.

[6] Vishnyakova M.A., Frolova N., Frolov A. (2023). Drought Stress Response in Guar (*Cyamopsis tetragonoloba* (L.) Taub): Physiological and Molecular Genetic Aspects. Plants.

[7] MacMillan J., Adams C.B., Trostle C., Rajan N. (2021). Testing the Efficacy of Existing USDA Rhizobium Germplasm Collection Accessions as Inoculants for Guar. Ind. Crop. Prod..

[8] Hinson P.O., Adams C.B. (2020). Quantifying Tradeoffs in Nodulation and Plant Productivity with Nitrogen in Guar. Ind. Crop. Prod..

[9] Morris J.B. (2010). Morphological and Reproductive Characterization of Guar (*Cyamopsis tetragonoloba*) Genetic Resources Regenerated in Georgia, USA. Genet. Resour. Crop Evol..

[10] Shrestha R., Adams C.B., Ravelombola W., MacMillan J., Trostle C., Ale S., Hinson P. (2021). Exploring Phenotypic Variation and Associations in Root Nodulation, Morphological, and Growth Character Traits among 50 Guar Genotypes. Ind. Crop. Prod..

[11] Suzaki T., Yoro E., Kawaguchi M. (2015). Leguminous Plants: Inventors of Root Nodules to Accommodate Symbiotic Bacteria. Int. Rev. Cell Mol. Biol..

[12] Khandelwal A., Sindhu S.S. (2012). Expression of 1-Aminocyclopropane-1-Carboxylate Deaminase in Rhizobia Promotes Nodulation and Plant Growth of Clusterbean (*Cyamopsis tetragonoloba* L.). Res. J. Microbiol..

[13] Venkateswarlu B., Rao A.V., Lahiri A.N. (1983). Effect of Water Stress on Nodulation and Nitrogenase Activity of Guar (*Cyamopsis tetragonoloba* (L.) Taub.). Proc. Plant Sci..

[14] Farooq S., Azam F. (2002). Molecular Markers in Plant Breeding-I: Concepts and Characterization. Pak. J. Biol. Sci..

[15] Caetano-Anollés G. (1997). Molecular Dissection and Improvement of the Nodule Symbiosis in Legumes. Field Crops Res..

[16] Oladzad A., González A., Macchiavelli R., de Jensen C.E., Beaver J., Porch T., McClean P. (2020). Genetic Factors Associated With Nodulation and Nitrogen Derived From Atmosphere in a Middle American Common Bean Panel. Front. Plant Sci..

[17] Duran C., Appleby N., Clark T., Wood D., Imelfort M., Batley J., Edwards D. (2009). AutoSNPdb: An Annotated Single Nucleotide Polymorphism Database for Crop Plants. Nucleic Acids Res..

[18] Ravelombola W., Shi A., Weng Y., Mou B., Motes D., Clark J., Chen P., Srivastava V., Qin J., Dong L. (2018). Association Analysis of Salt Tolerance in Cowpea (*Vigna unguiculata* (L.) Walp) at Germination and Seedling Stages. Theor. Appl. Genet..

[19] Malani S., Ravelombola W., Adams C.B., Ibrahim A., Ale S. (2024). Evaluation of Nodule Traits in USDA Guar Genotype Accessions. Euphytica.

[20] Malani S., Ravelombola W., Manley A., Pham H. (2024). Genetic Diversity and Population Structure Analysis in Guar. Plants.

[21] Stanton-Geddes J., Paape T., Epstein B., Briskine R., Yoder J., Mudge J., Bharti A.K., Farmer A.D., Zhou P., Denny R. (2013). Candidate Genes and Genetic Architecture of Symbiotic and Agronomic Traits Revealed by Whole-Genome, Sequence-Based Association Genetics in Medicago Truncatula. PLoS ONE.

[22] Ksiazkiewicz M., Nazzicari N., Yang H., Nelson M.N., Renshaw D., Rychel S., Ferrari B., Carelli M., Tomaszewska M., Stawiński S. (2017). A High-Density Consensus Linkage Map of White Lupin Highlights Synteny with Narrow-Leafed Lupin and Provides Markers Tagging Key Agronomic Traits. Sci. Rep..

[23] Yu J., Pressoir G., Briggs W.H., Bi I.V., Yamasaki M., Doebley J.F., McMullen M.D., Gaut B.S., Nielsen D.M., Holland J.B. (2005). A Unified Mixed-Model Method for Association Mapping That Accounts for Multiple Levels of Relatedness. Nat. Genet..

[24] Liu X., Huang M., Fan B., Buckler E.S., Zhang Z. (2016). Iterative Usage of Fixed and Random Effect Models for Powerful and Efficient Genome-Wide Association Studies. PLoS Genet..

[25] Azeem A., Mai W., Ali R. (2024). Modeling Plant Height and Biomass Production of Cluster Bean and Sesbania across Diverse Irrigation Qualities in Pakistan’s Thar Desert. Water.

[26] Grigoreva E., Barbitoff Y., Changalidi A., Karzhaev D., Volkov V., Shadrina V., Safronycheva E., Ben C., Gentzbittel L., Potokina E. (2021). Development of SNP Set for the Marker-Assisted Selection of Guar (*Cyamopsis tetragonoloba* (L.) Taub.) Based on a Custom Reference Genome Assembly. Plants.

